# Evaluation of the efficacy of palonosetron for prevention of chemotherapy-induced nausea and vomiting in patients with gastric cancer treated with S-1 plus cisplatin

**DOI:** 10.1007/s10147-015-0916-2

**Published:** 2015-10-27

**Authors:** Katsunobu Oyama, Sachio Fushida, Masahide Kaji, Toshiya Takeda, Kazuhisa Yabushita, Hideaki Nezuka, Shinichi Kinami, Naotaka Kadoya, Yuki Takai, Yuji Tsukioka, Shigekazu Ohyama, Kunihiro Tsuji, Tomoya Tsukada, Jun Kinoshita, Takashi Fujimura, Tetsuo Ohta

**Affiliations:** Department of Gastroenterological Surgery, Kanazawa University, 13-1 Takara-machi, Kanazawa, Ishikawa 920-8641 Japan; Department of Surgery, Toyama Prefectural Central Hospital, Toyama, Japan; Department of Surgery, Public Central Hospital of Matto Ishikawa, Hakusan, Japan; Department of Surgery, Takaoka City Hospital, Takaoka, Japan; Department of Surgery, Yatsuo General City Hospital, Toyama, Japan; Department of General and Digestive Surgery, Kanazawa Medical University, Uchinada, Japan; Department of Surgery, Toyama Rosai Hospital, Uozu, Japan; Department of Gastroenterology, Keiju Medical Center, Nanao, Japan; Department of Surgery, Toyama City Hospital, Toyama, Japan; Department of Surgery, National Hospital Organization Kanazawa Medical Center, Kanazawa, Japan; Department of Gastroenterology, Ishikawa Prefectural Central Hospital, Kanazawa, Japan

**Keywords:** Palonosetron, Gastric cancer, CINV, Anorexia, QOL

## Abstract

**Purpose:**

The purpose of our study was to evaluate the efficacy of a new combination antiemetic therapy consisting of palonosetron, aprepitant, and dexamethasone in gastric cancer patients undergoing chemotherapy with S-1 plus cisplatin.

**Methods:**

This prospective, multi-institutional observational study assessed patient-reported nausea, vomiting, use of rescue therapy, change of dietary intake, and Functional Living Index-Emesis (FLIE) questionnaire results. The percentages of patients showing complete response (CR; no emesis and non-use of any rescue antiemetics) and complete protection (CP; no significant nausea and non-use of any rescue antiemetics), change of dietary intake, and impact of chemotherapy-induced nausea and vomiting on daily life during the overall (0–120 h after cisplatin administration), acute (0–24 h), and delayed (24–120 h) phases were examined. These findings were compared with our previous study, which used granisetron, aprepitant, and dexamethasone, to assess the relative effectiveness of palonosetron versus granisetron in combination antiemetic therapy.

**Results:**

Of the 72 included patients, 66 (91.6 %), 70 (97.2 %), and 50 (69.1 %) achieved CR, and 48 (66.7 %), 61 (84.7 %) and 49 (68.1 %) achieved CP during in the overall, acute, and delayed phases of cisplatin administration, respectively. Approximately half of the patients had some degree of anorexia. FLIE results indicated that 78.6 % of patients maintained their quality of life. Palonosetron was not superior to granisetron in combination antiemetic therapy.

**Conclusions:**

Three-drug combination antiemetic therapy with palonosetron, aprepitant, and dexamethasone was tolerable in gastric cancer patients undergoing treatment with S-1 plus cisplatin. The predominance of palonosetron to granisetron was not demonstrated in this study.

## Introduction

Chemotherapy-induced nausea and vomiting (CINV) is a common adverse event observed in patients with cancer who receive chemotherapy [1] and is one of the greatest fears of patients receiving chemotherapy [2, 3]. Inadequate control of CINV can lead to dehydration, nutritional deficiencies, and electrolyte imbalances, which may impair functional and mental activities and quality of life (QOL), increase the use of healthcare resources, and occasionally cause treatment delay or discontinuation [4–6]. Generally, CINV persists for approximately 5 days after administration of emetogenic antitumor agents. CINV occurring within the first 24 h has been defined as acute and that occurring after >24 h as delayed [7].

Corticosteroids have long been used as antiemetic agents to treat CINV [8], being effective for both acute and delayed emesis. The introduction of selective serotonin [5-hydroxytryptamine-3 (5-HT_3_)] receptor antagonists (RAs), such as ondansetron, dolasetron, and granisetron revolutionized the control of CINV. 5-HT_3_RAs are used to treat patients receiving moderate to highly emetogenic chemotherapy, with protective effects mainly in acute emesis. Although combinations of corticosteroids and 5-HT_3_RA have been standard for the management of CINV, >50 % of patients continue to vomit in response to highly emetogenic chemotherapy, such as high-dose cisplatin [9], suggesting that this combination antiemetic therapy prevents vomiting in the acute phase, but not in the delayed phase [10–12]. Delayed nausea also tends to be more severe and less responsive to antiemetic therapy than acute nausea.

Gastric cancer is one of the major causes of cancer deaths worldwide, and chemotherapy is the main treatment option for patients with advanced stage disease. A standard chemotherapeutic regimen for advanced gastric cancer consists of a combination of cisplatin plus fluoropyrimidine, which induces CINV. A large phase III trial of S-1, an orally administrated 5-fluorouracil analog, plus cisplatin in patients with advanced gastric cancer who received combinations of corticosteroids and 5-HT_3_RAs for management of CINV found that emesis occurred in 36 % of these patients and nausea in 67 % [13].

Recently, two novel anti-CINV agents have become available, the neurokinin-1 (NK_1_) RA aprepitant and the new generation 5-HT_3_RA palonosetron, both of which were found to prevent delayed-phase CINV. An observational study testing the combination of aprepitant, granisetron and dexamethasone in patients receiving chemotherapy with S-1 plus cisplatin for gastric cancer found that complete response (CR; no emesis and non-use of any rescue antiemetics) rates overall and during the acute and delayed phases of chemotherapy administration were 88.7, 98.1, and 88.7 %, respectively, and complete protection (CP; no significant nausea and non-use of any rescue antiemetics) rates during the three phases of chemotherapy were 67.9, 96.2, and 67.9 %, respectively [14]. Similarly, a study of triplet antiemetic treatment with a corticosteroid, a first generation 5-HT_3_RA and NK_1_RA reported that this largely alleviated acute and some delayed emesis, but found that delayed nausea rates were unacceptably high [15].

Palonosetron is a new generation 5-HT_3_RA, differing from first generation 5-HT_3_RAs in its pharmacokinetic and pharmacodynamic profiles, and may be uniquely suited to treat both early and delayed CINV. Four phase III clinical trials found that CINV prevention rates were higher with palonosetron than with first-generation 5-HT_3_RAs [16–19]. Palonosetron has a longer elimination half-life (*t*_½_) and a greater receptor-binding affinity than first-generation 5-HT_3_RAs [20]. Moreover, it has been shown to trigger 5-HT_3_ receptor internalization and prolong inhibition of receptor function [21]. Moreover, mechanistic studies using palonosetron and an NK_1_RA suggest interactions between the 5-HT_3_ receptor and NK_1_ receptor neurotransmitter pathways [22].

When compared with other 5-HT_3_RAs, palonosetron was found to significantly reduce subjective sensations of nausea [23]. A pooled analysis of phase III clinical trials comparing palonosetron with first-generation 5-HT_3_RAs found that palonosetron was superior in controlling nausea [24] and may therefore be superior in controlling delayed nausea.

This prospective observational study assessed the antiemetic efficacy and tolerability of palonosetron, combined with dexamethasone and aprepitant, in patients with advanced gastric cancer receiving S-1 plus cisplatin chemotherapy. By performing this study in patients similar to those who received triplet antiemetic therapy with granisetron, dexamethasone and aprepitant [14], we were able to compare the relative effectiveness of palonosetron and granisetron in combination antiemetic therapy for patients with gastric cancer.

## Methods

### Design

This was a multi-institutional, prospective, observational, non-comparative study involving 20 institutions of the Digestive Disease Support Organization (DDSO). All patients provided written informed consent, and the study protocol was approved by the institutional review board at each participating center. Moreover, the study was performed in accordance with the principles of the Declaration of Helsinki (Clinical trial ID: UMIN000009016).

### Eligibility criteria

The study involved high or moderately emetogenic, chemotherapy-naive patients scheduled to receive their first course of chemotherapy with S-1 (80 mg/m^2^) and cisplatin (60 mg/m^2^) for pathologically confirmed gastric cancer. All patients were aged ≥20 years and had an Eastern Cooperative Oncology Group (ECOG) Performance Status of 0–2. Patients with any vomiting, retching, or nausea [National Cancer Institute (NCI) ≥grade I] in the 24 h before the start of chemotherapy and those using any drug with potential antiemetic efficacy 48 h before chemotherapy were excluded. In addition, patients receiving radiation therapy to the abdomen or pelvis less than 1 week before treatment, those with a symptomatic primary or metastatic central nervous system malignancy, those at risk of vomiting for other reasons (e.g., epilepsy, active peptic ulcer, and gastrointestinal obstruction), and those with any uncontrolled disease other than malignancy that may pose an unwarranted risk, as determined by the investigator, were also excluded.

### Chemotherapy

All patients received S-1 plus cisplatin therapy as described in the SPIRITS trial [13], the standard chemotherapeutic regimen for advanced gastric cancer in Japan. S-1 (80 mg/m^2^) was administered orally twice daily for the first 3 weeks of each 5-week cycle. Cisplatin was administered as an intravenous infusion of 60 mg/m^2^ on day 8 of each cycle.

### Antiemetic treatment

Patients received the combination of antiemetics recommended in the 2010 Japanese Society of Clinical Oncology (JSCO) Guidelines for Antiemetics in Oncology [25]. On day 1, all patients received oral aprepitant 125 mg 60 min before cisplatin infusion plus intravenous dexamethasone 9.9 mg and intravenous palonosetron 0.75 mg 30 min before cisplatin infusion. On days 2 and 3, patients received oral aprepitant 80 mg once daily each morning and oral dexamethasone 8 mg bid, and on day 4 patients received oral dexamethasone 8 mg bid. Patients were also prescribed a rescue antiemetic, to be used only when nausea and vomiting developed during the 120-h observation period.

### Response definitions

The observation period was divided into three distinct phases after injection of cisplatin—acute (0–24 h), delayed (24–120 h), and overall (0–120 h). During each 120-h assessment period, patients were required to maintain a diary and record the number and timing of any episodes of vomiting or retching; the frequency and timing of use of rescue antiemetics; and the degree of nausea using a 4-point categorical scale (0, none; 1, mild; 2, moderate; 3, severe). Changes in dietary intake were recorded by patients every day on days 1–5 as percent volume of diet after initiation of chemotherapy compared with before. Patients also completed the FLIE questionnaire once per day from days 1–5; this questionnaire captured information about the effect of CINV on their daily lives.

The FLIE is a patient-completed multidimensional questionnaire that evaluates the QOL [26]. The Japanese version of the FLIE, which was used in this study, has been reported useful in assessing the impact of CINV on the QOL of Japanese patients [27]. The FLIE questionnaire contains a validated 18-item visual analog scale (VAS)-based, patient-reported outcome measure that captures information about the effect of CINV on the daily lives of the patients. FLIE has separate domains for the impact of nausea and vomiting on the daily function of patients. Each item is scored from 7 (not at all) to 1 (a great deal). Cut-offs for a minimal or no impact of CINV on daily life included an average score of >6 points, a total score of >108 of the maximum possible 126 points, and a score on each domain of >54.

The primary endpoint was the proportion of patients achieving CR during the overall study phase. No vomiting was defined as no vomiting, retching, or dry heaves. Secondary endpoints included the rate of CP. No significant nausea was defined as nausea scores of 0 and 1, and as nausea that does not interfere with normal patient activities; changes in dietary intake; absence of vomiting and no nausea; and the impact of CINV on daily life, as measured by the FLIE, during the overall, acute, and delayed phases. Safety was evaluated based on physical examinations, including vital signs, routine clinical laboratory tests, and adverse event reporting. Toxicity grades were assessed using the NCI Common Terminology Criteria for Adverse Events (NCI-CTCAE) v4.0. (http://ctep.cancer.gov/forms/CTCAEv4.pdf). Any adverse events were determined by the investigator to be possibly, probably, or definitively related to the study drug.

### Statistical analysis

In our previous study, CR was observed in 88.7 % and CP in 67.9 % of patients. To realize an equivalent antiemetic effect with palonosetron, the expected CR rate was set at 89 %, the threshold CR rate at 77 % and the expected CP rate at 80 %. Using these parameters, we calculated that an estimated sample size of 63 subjects was required to provide a power of 80 %, assuming a normal equation method and an overall significance level of 0.05. Assuming that approximately 15 % of subjects would be withdrawn or drop out, the target sample size was set at 75 subjects. Rates of CR and CP, food intake and impact on daily life in this study were compared with those in our previous [14] using chi-squared tests. All statistical analyses were performed using the Statistical Package for Social Sciences (SPSS) software version 11.0.

## Results

### Patient characteristics

Between November 2012 and May 2014, 75 patients were enrolled at 14 centers in Japan. Of these, 72 satisfied the eligibility criteria; the three patients who were excluded did not receive cisplatin injections. The baseline demographic and clinical characteristics of the patients are described in Table 1. Of the 72 patients, 58 (80.6 %) were male, and the median age of the patients was 65 years.

**Table 1:** Patient demographic and clinical characteristics

Characteristics		No. of patients
All		72
Age (years; 50–81, median 65)	≤65:≥66	34:38
Gender	Male:female	58:14
ECOG performance status	0:1	47:25
Clinical stage of gastric cancer (TNM)	II:III:IV:recurrence	0:20:37:15
Alcoholic drinks	None:seldom:almost daily	49:4:19
History of chemotherapy	Negative:positive	7:65
History of CINV	Negative:positive	72:0
History of morning sickness	Negative:positive	71:1
History of motion sickness	Negative:positive	71:1

### Antiemetic outcomes

**Fig. 1:**
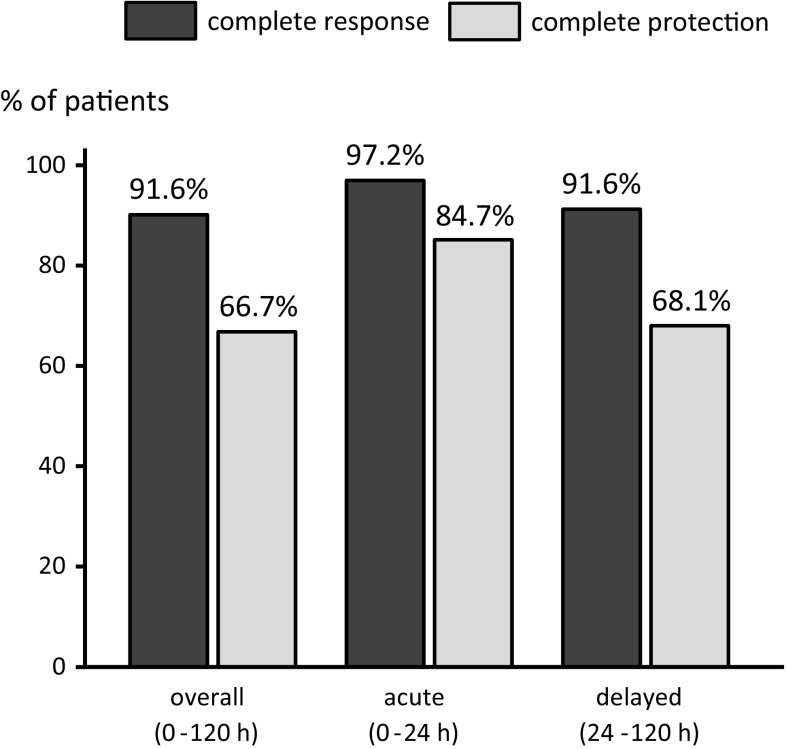
Percentage of patients with complete response (CR) and complete protection (CP). CR for the overall, acute, and delayed phases was achieved in 91.6, 97.2, and 91.6 % of patients, respectively. CP rates for the overall, acute, and delayed phases were 66.7, 84.7, and 68.1 %, respectively

Antiemetic outcomes are shown in Fig. 1 and Table 2. CR during the overall, acute, and delayed phases of cisplatin administration was achieved by 66 (91.6 %), 70 (97.2 %) and 66 (91.6 %) patients, respectively, and CP during these phases was achieved by 48 (66.7 %), 61 (84.7 %), and 49 patients (68.1 %), respectively.

**Table 2:** Percentage of patients achieving efficacy endpoints

	Overall (0–120 h)	Acute (0–24 h)	Delayed (24–120 h)
Complete response (%)	91.6	97.2	91.6
Complete protection (%)	66.7	84.7	68.1

### Assessment of the QOL

Of the 72 eligible patients, 70 completed the FLIE questionnaires (Table 3). Over the 5 day study period, 55 patients (78.6 %) had total FLIE scores of >108, with 54 (77.1 %) having a nausea domain score of >54 and 64 (91.4 %) having a vomiting domain score of >54, indicating that CINV had minimal or no impact on daily life. During the acute phase, 62 patients (88.6 %) had total FLIE scores, 63 (90.0 %) had nausea domain scores and 68 (97.2 %) had vomiting domain scores indicating that CINV had minimal or no impact on daily life. During the delayed phase, 55 patients (78.6 %) had total FLIE scores, 54 (77.1 %) had nausea domain scores and 66 (94.3 %) had vomiting domain scores indicating that CINV had minimal or no impact on daily life.

**Table 3:** QOL assessment based on FLIE questionnaire

FLIE item	Overall (0–120 h)			Acute (0–24 h)			Delayed (24–120 h)		
	No. of patients		%	No. of patients		%	No. of patients		%
	Total	NIDL		Total	NIDL		Total	NIDL	
FLIE total score	70	55	78.6	70	62	88.6	70	55	78.6
Nausea domain total score	70	54	77.1	70	63	90.0	70	54	77.1
Vomiting domain total score	70	64	91.4	70	68	97.2	70	66	94.3

*NIDL* no or minimal impact on daily life, defined as domain FLIE score of >54 and total FLIE score of >108

### Dietary intake

**Fig. 2:**
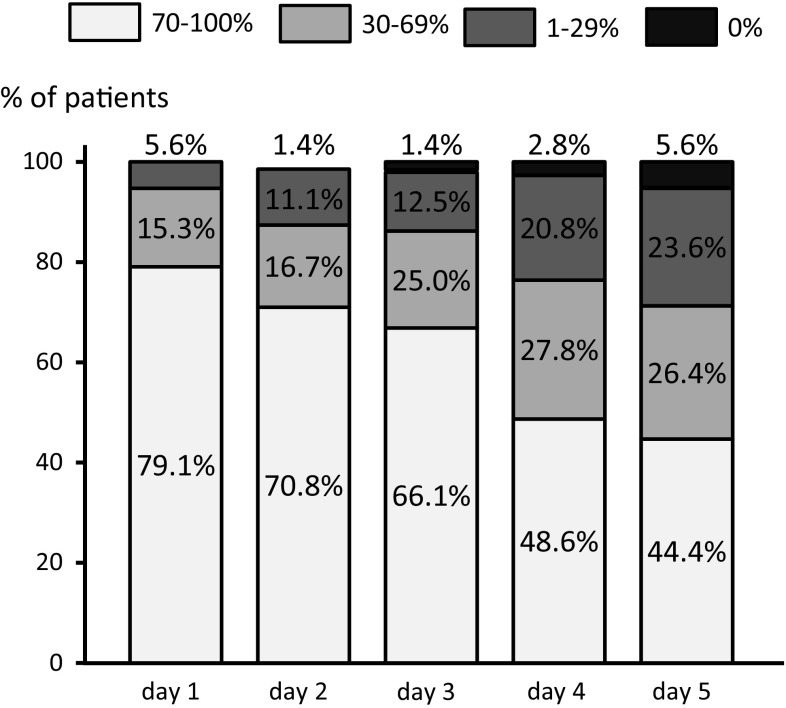
Decrease of diet intake compared with before chemotherapy period. Approximately half of the patients experienced some degree of anorexia; the decrease in oral intake was predominant in the delayed phase

Approximately half of the patients had some degree of anorexia, with the decrease in oral intake being more predominant during the delayed phase (Fig. 2). The volume of dietary intake was reduced by half in 30 % of patients. Additionally, 5 % of these patients could not consume any food or beverage during the delayed phase.

### Comparison between these results and those of our previous study

A comparison of CR, CP, diet intake and impact on daily life in this study and that of our previous study evaluating the combination of aprepitant, granisetron and dexamethasone as an antiemetic in advanced cancer patients treated with cisplatin and S-1 showed no significant differences in any of these parameters (Table 4).

**Table 4:** Comparison of efficacy outcomes with those of a previous study assessing the combination of granisetron, aprepitant, and dexamethasone

	Present study (palonosetron/aprepitant/granisetron) (%)	Previous study^a^ (granisetron/aprepitant/granisetron) (%)
Complete response		
Acute	97.2	98.1
Delayed	91.6	88.7
Overall	91.6	88.7
Complete protection		
Acute	84.7	96.2
Delayed	68.1	67.9
Overall	66.7	67.9
Reduction of diet intake		
Acute	20.9	18.8
Delayed	55.6	54.0
Overall	55.6	54.0
Impact on daily life (FLIE)		
Acute	88.6	98.0
Delayed	78.6	79.5
Overall	78.6	79.5

^a^From Ref. [14]

### Safety

Overall, antiemetic therapy was well tolerated. Adverse events considered by the investigator to be possibly, probably, or definitely related to the study drug included anorexia in nine (12.5 %) patients, diarrhea in six (8.3 %), and hiccups and constipation in one (1.4 %) each. No serious adverse events appeared related to the study drug.

## Discussion

Palonosetron, a new 5-HT_3_RA, first became available in 2003, the same year as aprepitant. Standard antiemetic therapy with corticosteroid and a first-generation 5-HT_3_RA provided significant advances in controlling acute emesis, but provided minimal benefit against delayed emesis. Palonosetron has a stronger binding affinity to its receptor and a longer plasma-elimination half-time than first generation 5-HT_3_RAs. As CINV can persist for several days, a longer acting 5-HT_3_RA may be valuable in its treatment. Clinical trials have shown that palonosetron was effective in preventing both acute and delayed CINV [15–18]. Two phase III randomized trials in patients receiving moderately emetogenic chemotherapy showed that monotherapy with palonosetron better prevented delayed phase emesis than ondansetron [15] or dolasetron [16]. Moreover, palonosetron was found to be superior to ondansetron in patient receiving highly emetogenic chemotherapy [17]. The most noteworthy differences between palonosetron and first-generation 5-HT_3_RAs occurred during the delayed phase. Although the latter two trials allowed dexamethasone pre-treatment at the investigator’s discretion, only one of these trials [17] found that concomitant dexamethasone was associated with antiemetic effects. A phase III trial comparing the anti-CINV effects of palonosetron plus dexamethasone versus granisetron plus dexamethasone in patients receiving highly emetogenic chemotherapy found that palonosetron was non-inferior to granisetron during the acute phase and superior to granisetron during the delayed phase [18]. A pooled-analysis of phase III trials comparing palonosetron with first-generation 5-HT_3_RAs in combination with corticosteroid showed that palonosetron was associated with higher rates of CR, CP, and absence of emesis and nausea [28]. Nevertheless, another meta-analysis found that the superiority of palonosetron was unclear in trials in which patients were administered dexamethasone [29]. At present, therefore, the superiority of palonosetron when combined with corticosteroid remains unclear.

Triplet antiemetic therapy, involving a 5-HT_3_RA, a corticosteroid and an NK_1_RA, is now standard for patients receiving highly emetogenic chemotherapy. It is unclear, however, whether palonosetron is more effective against delayed emesis when administered with an NK_1_RA. Several single-arm studies found that palonosetron had additional antiemetic efficacy when added to aprepitant and dexamethasone in patients with gynecological, head/neck and lung cancer [29–31]. Only one phase III trial, the TRIPLE study, compared granisetron and palonosetron added to basal antiemetic therapy with NK_1_RA and corticosteroid, finding that palonosetron was superior to granisetron in preventing delayed emesis and nausea [32]. It also remains unclear whether palonosetron and NK_1_RA act synergistically. In a recent report with gynecological cancer patients treated with moderately emetogenic chemotherapy including paclitaxel and carboplatin, the antiemetic effect of triplet therapy with palonosetron plus aprepitant plus dexamethasone was equivalent with triplet therapy with granisetron plus aprepitant plus dexamethasone [33]. The trial described here found that triple combination antiemetic therapy with palonosetron, aprepitant and dexamethasone for delayed emesis was well tolerated, but that the addition of palonosetron to aprepitant and dexamethasone was no more effective than the addition of granisetron to aprepitant and dexamethasone. However, the prolonged antiemetic effect of palonosetron may have been masked when administered with other antiemetic agents, including an NK_1_RA.

The discrepancy between our results and those of the TRIPLE trial may be due to the efficacy of previous triplet therapy with granisetron, dexamethasone and aprepitant being sufficient for gastric cancer patients treated with S-1 plus cisplatin, masking any additional effects of palonosetron. In fact, the CINV control rates in our studies were superior to those of other studies in patients with different types of cancer receiving several chemotherapeutic regimens.

Medical economics is an important issue in clinical practice and must be balanced with the results of evidence-based medicine. The drug costs of antiemetics including palonosetron was JPY 27.653 and JPY 16,808 with granisetron per course of chemotherapy. In the present study, the drug replacement from granisetron to palonosetron is not worth the cost.

This study had several limitations. First, the study cohort was limited to patients with gastric cancer receiving an initial course of S-1 plus cisplatin chemotherapy. Patients with other types of cancer receiving other chemotherapeutic regimens may differ in response to triple antiemetic regimens that include palonosetron. Second, the number of enrolled patients was relatively small, and the percentages of female (14/72) and younger (median age 65 years old) patients were low. This is important as female sex and younger age are risk factors for emesis. Third, our study was an observational study, such that patients and caregivers were not blinded to the therapeutic regimens. Randomized controlled trials with a greater number of subjects are required to verify the findings presented here.

In conclusion, the tolerability of triple combination antiemetic therapy with palonosetron, aprepitant and dexamethasone was satisfactory. Nevertheless, we did not observe an additional effect of palonosetron in patients with gastric cancer treated initially with S-1 plus cisplatin. The optimal antiemetic therapy may differ in different types of cancer and in patients receiving different chemotherapeutic regimens. New strategies are needed to further improve nausea and loss of appetite in cancer patients receiving chemotherapy.
